# When It Looks Like a Duck and Walks Like a Duck: Importance of DOTATATE PET Imaging in Assessing Putative Sphenoid Wing Meningioma for Stereotactic Radiosurgery

**DOI:** 10.7759/cureus.98574

**Published:** 2025-12-06

**Authors:** Afua Ofori-Darko, Shearwood McClelland III

**Affiliations:** 1 Department of Radiation Oncology, University of Oklahoma Health Sciences Center, Oklahoma City, USA; 2 School of Medicine, Case Western Reserve University School of Medicine, Cleveland, USA; 3 Department of Neurological Surgery, University of Oklahoma Health Sciences Center, Oklahoma City, USA

**Keywords:** dotatate, ga-68 dotatate scan, meningioma diagnosis, neuro mri, neuro-ophthalmology, neurosurgery, photon stereotactic radiosurgery, radiation oncology education, sphenoid ridge meningioma, venous varix

## Abstract

MRI is central to evaluating skull-base lesions, but morphologic features alone can misclassify vascular anomalies as neoplasms. A 27-year-old man presented with left monocular diplopia and an enhancing dural-based lesion along the lateral-superior wall of the left cavernous sinus, read as a sphenoid-wing meningioma. He was evaluated for stereotactic radiosurgery. Incorporation of ^68^Ga-DOTATATE PET demonstrated no somatostatin receptor avidity, prompting neuroradiology re-review of prior angiography and subsequent orbital CT that supported a cavernous-sinus venous varix. Stereotactic radiosurgery was withheld, and the patient remained clinically stable on observation with a second DOTATATE PET confirming non-avidity. This case highlights the value of DOTATATE PET as a complementary study for indeterminate skull-base lesions, particularly when tissue diagnosis is unavailable, to prevent inappropriate intervention.

## Introduction

Meningiomas are responsible for approximately one-third of all primary intracranial neoplasms and, if diagnosed on a radiographic basis, are frequently encountered when there is a homogeneously enhancing dural-based lesion with a dural tail present in the vicinity of the skull base [1]. Vascular abnormalities of the cavernous sinus may enhance and simulate these features, creating a risk of misdiagnosis if decisions rely on MRI alone. ^68^Ga-DOTATATE binds somatostatin receptor subtype 2, which is strongly expressed by most meningiomas, and has proven useful for diagnosis, radiotherapy planning, and follow-up in this disease [2]. We present a case in which DOTATATE PET averted stereotactic radiosurgery (SRS) by rediagnosing a presumed meningioma as a venous varix.

## Case presentation

**Figure 1:**
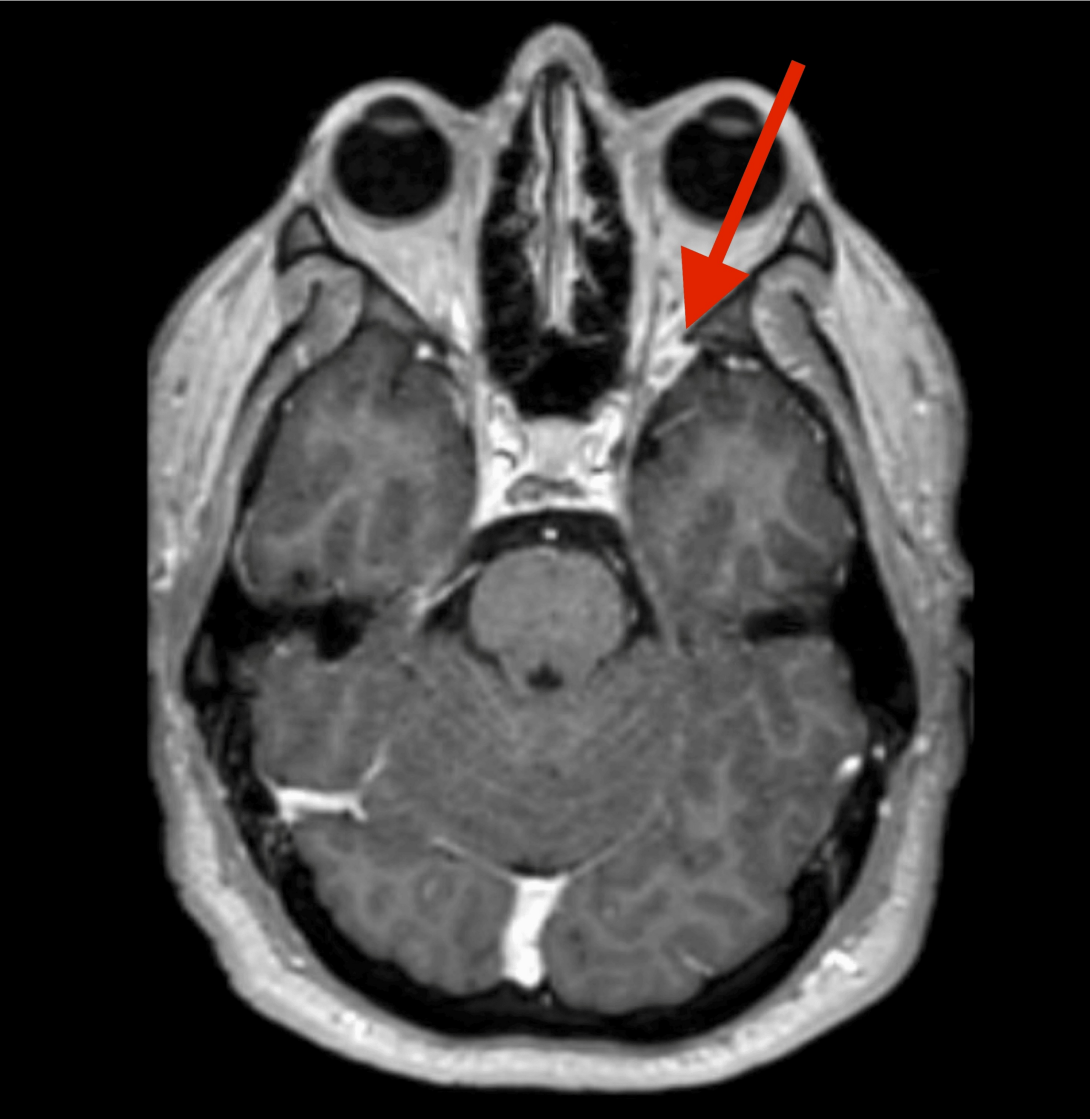
Axial T1-weighted post-contrast MRI demonstrating a homogeneously enhancing dural-based lesion along the left cavernous sinus with mild sphenoid-wing extension, initially interpreted as a sphenoid-wing meningioma.

A 27-year-old man presented with a seven-month history of left monocular diplopia that resolved when viewing with the right eye. He remained able to drive and function at work. Neuro-ophthalmologic examination found left hypertropia without trochlear nerve palsy. Contrast-enhanced brain MRI showed a homogeneously enhancing dural-based lesion along the lateral-superior wall of the left cavernous sinus with mild sphenoid-wing extension, consistent with a putative sphenoid-wing meningioma (Figure 1), and the patient was referred to neurosurgery and radiation oncology for further evaluation. Neurosurgery suggested resection, observation, or SRS. The patient wished to avoid open surgery while restoring normal vision. 

**Figure 2:**
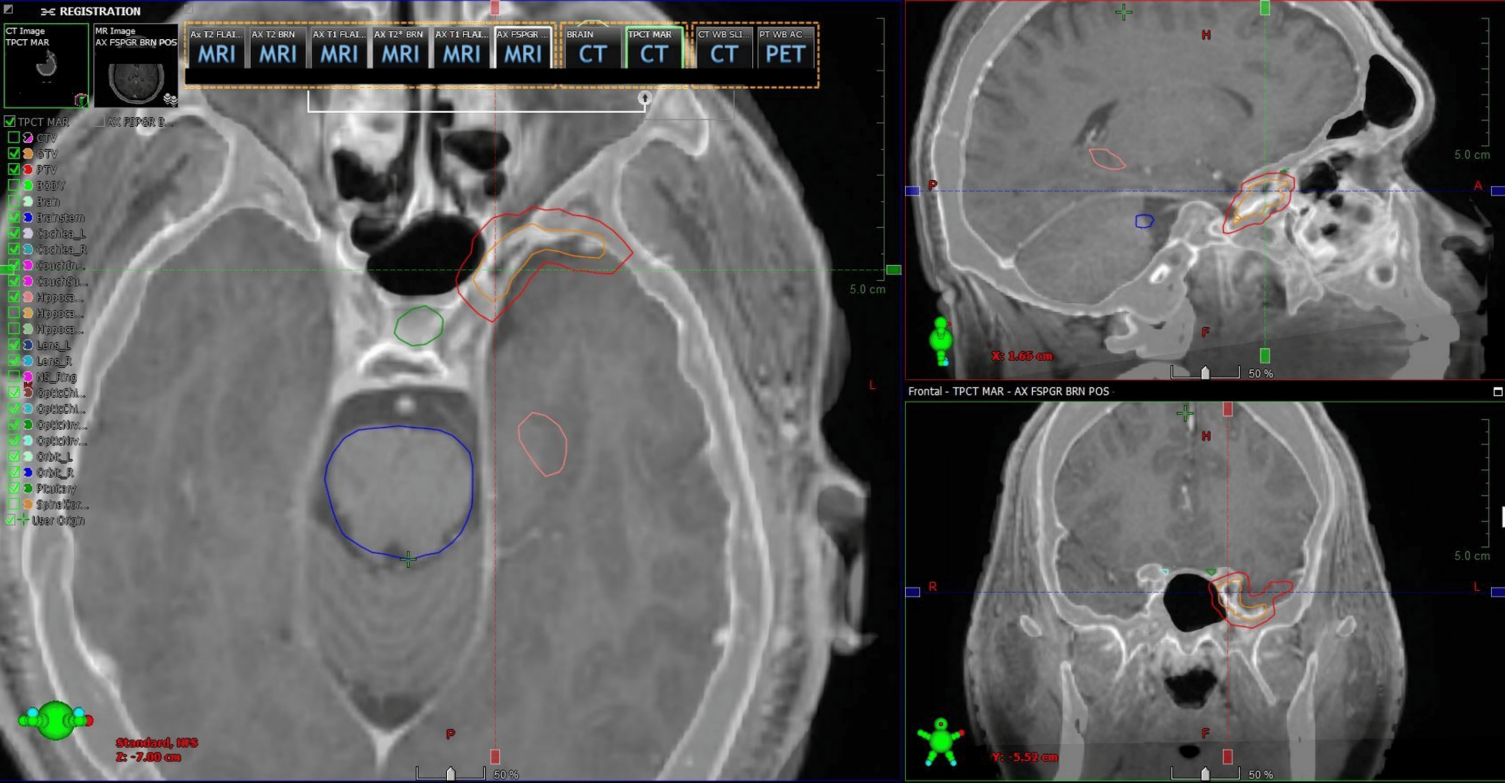
CT/MRI fusion before obtaining 68Ga-DOTATATE PET. Co-registered T1 post-contrast MRI and contrast-enhanced CT images showing accurate alignment of the left cavernous sinus lesion and adjacent skull base structures for subsequent molecular imaging registration.

Radiation oncology recommended fractionated linear-accelerator SRS, given the cavernous-sinus location and proximity to the optic apparatus and internal carotid artery, and ordered a repeat planning MRI and ^68^Ga-DOTATATE PET/CT before treatment planning. The CT/MRI fusion used for treatment planning is shown in Figure 2. A CT angiogram was additionally obtained to exclude cerebrovascular pathology.

**Figure 3:**
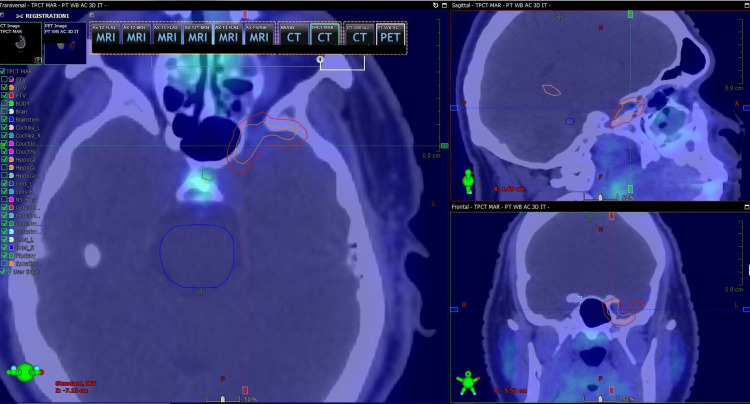
Treatment-planning CT fused with 68Ga-DOTATATE PET showing absence of tracer uptake within the contoured lesion at the left cavernous sinus, supporting a venous varix rather than a meningioma.

At initial presentation, CT angiography suggested a vascular structure contiguous with the cavernous sinus. Two weeks later, a repeat MRI again demonstrated the enhancing lesion abutting the left cavernous sinus. Shortly thereafter, DOTATATE PET/CT showed no radiotracer uptake within the lesion (Figure 3). Within the following week, neuroradiology re-reviewed the CT angiogram and favored a venous varix arising from the left cavernous sinus rather than a solid extra-axial tumor. The next week, an orbital CT corroborated a lack of soft-tissue mass with a venous varix. Approximately two weeks later, a follow-up with neuro-ophthalmology documented a stable examination. Three months later, repeat DOTATATE PET again showed no avidity. Given the concordance of DOTATATE PET, CT angiography, MRI, and orbital CT, SRS was not performed. At follow-up 11 months from symptom onset, the patient reported no new neurologic symptoms and continued under observation by neuro-ophthalmology. 

## Discussion

This case demonstrates how a vascular lesion in the cavernous sinus can mimic meningioma on contrast-enhanced MRI and how functional imaging with DOTATATE PET can resolve this ambiguity. The cavernous sinus contains venous channels and the cavernous segment of the internal carotid artery in close association with cranial nerves III through VI. Venous varices and venous ectasias in this region may appear as smoothly enhancing foci that abut the dura and can be indistinguishable from meningioma on morphology alone [3]. Because biopsy involving the cavernous sinus carries a significant risk, clinicians often depend on imaging to guide management.

Somatostatin receptor imaging provides a biologic signature that complements MRI. Multiple studies have shown high sensitivity of DOTATATE or DOTATOC for identifying meningiomas because of the near ubiquitous and intense expression of somatostatin receptor subtype 2 in these tumors [4-6]. In a prospective study, Maffione et al. demonstrated DOTATATE PET/CT to accurately identify meningioma tissue with high accuracy and improve detection of skull-base disease compared with MRI, particularly at the cavernous sinus and skull base, where postsurgical change or vascular structures complicate interpretation [4]. Sulzbacher et al. reported strong lesion-to-background contrast on ^68^Ga-DOTATOC PET/CT with high meningioma sensitivity across different anatomic sites [5]. Jin and colleagues showed that integrating DOTATATE PET into target delineation for radiation therapy significantly altered gross tumor volumes, most often at the skull base, where MRI alone was limited [6]. Mahase et al. described the feasibility and added benefit of hybrid ^68^Ga-DOTATATE PET/MRI for treatment planning and again reinforced the role of receptor-targeted imaging in meningioma management [2].

In the present case, the absence of DOTATATE avidity argued strongly against meningioma and prompted critical re-evaluation of the vascular anatomy. Subsequent multimodality review upon re-evaluation converged on a diagnosis of venous varix, which is optimally treated by observation rather than with SRS. Proceeding with SRS on the basis of MRI alone would have exposed a young patient to risks, including cranial neuropathy, optic pathway damage, radiation necrosis, secondary cancer formation, and unintended effects on vascular structures without the potential for oncologic benefit. This outcome emphasizes how functional imaging can prevent iatrogenic damage.

Routine use of DOTATATE PET is particularly important if diagnosis relies on imaging rather than tissue, if the lesion is in the cavernous sinus or other skull-base locations where morphology is confounded by venous structures, and if treatment entails focal therapy (i.e., high-dose focal radiation and/or operative resection). Although availability and reimbursement can vary by region, growing evidence supports DOTATATE PET as a decision-influencing adjunct that improves diagnostic confidence, refines target definition, and can change management in a meaningful fraction of cases [4-6]. In practice, early ordering by radiology, radiation oncology, or neurosurgery can help prevent delays and decrease the likelihood of near-miss events during treatment planning.

## Conclusions

This case illustrates the importance of routine incorporation of DOTATATE PET imaging for SRS planning, particularly for lesions lacking a confirmatory tissue diagnosis, which otherwise “look like a duck and walk like a duck.” In this instance, DOTATATE PET altered management in a patient with an enhancing cavernous sinus lesion initially presumed from MRI to be a sphenoid wing meningioma. The DOTATATE PET scan demonstrated a lack of somatostatin receptor avidity, leading to reclassification of the lesion as a venous varix. Subsequent multimodality review confirmed the vascular diagnosis. As a result, SRS was avoided, and the patient remained clinically stable under observation. As DOTATATE PET remains a relatively new imaging modality to assess meningioma, even in multidisciplinary patient management, this modality may not be acquired unless initiated by radiation oncology, as in this case. Consequently, any suspected meningioma without tissue diagnosis should receive DOTATATE PET evaluation as standard-of-care before consideration of operative or SRS intervention.
